# Intra-Amniotic Administration of Cashew Nut (*Anacardium occidentale* L.) Soluble Extract Improved Gut Functionality and Morphology In Vivo (*Gallus gallus*)

**DOI:** 10.3390/nu15102378

**Published:** 2023-05-19

**Authors:** Talitha Silva Meneguelli, Nikolai Kolba, Arundhati Misra, Ana Paula Dionísio, Ana Claudia Pelissari Kravchychyn, Bárbara Pereira Da Silva, Hercia Stampini Duarte Martino, Helen Hermana Miranda Hermsdorff, Elad Tako

**Affiliations:** 1 Department of Food Science, Cornell University, Ithaca, NY 14850, USA; talithasilvameneguelli@gmail.com (T.S.M.); nk598@cornell.edu (N.K.); am2657@cornell.edu (A.M.); 2 Embrapa Agroindústria Tropical—CNPAT, Fortaleza 60511-110, CE, Brazil; ana.dionisio@embrapa.br; 3 Department of Nutrition and Health, Universidade Federal de Vicosa, Viçosa 36570-900, MG, Brazil; ana.pelissari@ufv.br (A.C.P.K.); barbara.p.silva@ufv.br (B.P.D.S.); hercia@ufv.br (H.S.D.M.); helenhermana@ufv.br (H.H.M.H.)

**Keywords:** cashew nut, *Anacardium occidentale* L., *Gallus gallus*, gut health

## Abstract

Cashew nuts are rich in dietary fibers, monounsaturated fatty acids, carotenoids, tocopherols, flavonoids, catechins, amino acids, and minerals that offer benefits for health. However, the knowledge of its effect on gut health is lacking. In this way, cashew nut soluble extract (CNSE) was assessed in vivo via intra-amniotic administration in intestinal brush border membrane (BBM) morphology, functionality, and gut microbiota. Four groups were evaluated: (1) no injection (control); (2) H_2_O injection (control); (3) 10 mg/mL CNSE (1%); and (4) 50 mg/mL CNSE (5%). Results related to CNSE on duodenal morphological parameters showed higher Paneth cell numbers, goblet cell (GC) diameter in crypt and villi, depth crypt, mixed GC per villi, and villi surface area. Further, it decreased GC number and acid and neutral GC. In the gut microbiota, treatment with CNSE showed a lower abundance of *Bifidobacterium*, *Lactobacillus*, and *E. coli*. Further, in intestinal functionality, CNSE upregulated aminopeptidase (AP) gene expression at 5% compared to 1% CNSE. In conclusion, CNSE had beneficial effects on gut health by improving duodenal BBM functionality, as it upregulated AP gene expression, and by modifying morphological parameters ameliorating digestive and absorptive capacity. For intestinal microbiota, higher concentrations of CNSE or long-term intervention may be necessary.

## 1. Introduction

The nutritional benefits of nuts to human health are well documented, primarily in the context of cardiovascular health [1,2], glycemic control [3], lipid metabolism [4,5,6], and antioxidants effects, as nuts provide numerous nutrients that play a key role in the possible prevention of cardiometabolic diseases, including favorable fatty acid (FA) profiles such as unsaturated FAs, dietary fiber, antioxidants, minerals (magnesium, calcium, manganese, selenium, and potassium), vitamins E, K, and B-vitamins, and phytonutrients (lutein, phytosterols, and flavonoids) [7,8]. The link between health, disease, and diversity of the gut microbiome is known since disruption of the gut microbiome, or dysbiosis, is associated with an increased risk of cardiometabolic diseases, such as metabolic syndrome (MetS), type 2 diabetes mellitus (T2DM), development of systematic inflammation, and cardiovascular disease (CVD) [9,10,11,12]. However, there are only a few studies that discuss the impact of nuts (only including almonds, walnuts, hazelnuts, and pistachio) on the intestinal microbiota [13,14]. The beneficial modification of microbiota composition is a promising approach for improving intestinal and overall health, as dietary fibers and phytochemicals that reach the proximal colon, such as those found in nuts, provide substrates to maintain the health and diversity of the microbiota [15]. Nuts are one of the most fiber-rich foods [16,17], and evidence shows a correlation between nut consumption and changes in the gut microbiome, indicating the prebiotic properties of the tree nut family [18]. However, the mechanisms by which nuts offer their prebiotic effects on microbial diversity are not fully understood [18]. 

In 2020/2021 (5.3 million tons), the global production of nuts grew 15% compared to 2011/2012 and 65% more than the previous decade. Furthermore, the global production of cashew nuts almost doubled in 2020/2021 (836,940 tons) compared to 2010/2011 (469,079 tons) [19], occupying the third position (16%), only behind almonds (31%), walnuts, and pistachios (19% each) [19]. Moreover, cashew nuts are the third most consumed (18%) nuts in the world, behind only almonds (30%) and walnuts (20%) [19]. The cashew tree (*Anacardium occidentale* L.) belongs to the *Anacardiaceae* family and is mainly in tropical regions [20]. The cashew nut is native to Northeast Brazil, present in the Cerrado biome [21,22,23]. It is cultivated primarily in India, Vietnam, Côte d’Ivoire, Guinea-Bissau, Tanzania, Benin, Brazil, and other countries in East and West Central Africa and Southeast Asia [23]. 

*Anacardium occidentale* L. contains various nutrients and bioactive compounds, including monounsaturated fatty acids, β-carotene, lutein, zeaxanthin, vitamin E as tocopherols, dietary fibers, flavonoids, catechins, amino acids, minerals, anacardic acids, cardanols, cardols, and phytosterols [21,24,25]. Most of these bioactive compounds present in cashew nuts play an important role in gut health as demonstrated by studies; catechins can modulate microbiota [26], selenium can affect gut microbial colonization avoiding health damage associated with dysbiosis [27], β-carotene can increase tight junctions expression (ZO-1, ZO-2, occludin), MUC-2 and MUC-3 expression, and increase villus height and villi height/crypt depth ratio [28,29], and lutein can decrease crypt depth [30]. Furthermore, flavonoids can modulate biomarkers relevant to intestinal inflammation [31]. In addition, cashews appear to have prebiotic properties, as an in vitro study demonstrated that defatted cashew nut flour had a prebiotic effect, increasing beneficial bacteria such as *Bifidobacterium* [32]. Non-bio-accessible nutrients from cashew nuts, such as polyphenols, polysaccharides, and dietary fiber, have prebiotic properties [33,34]. The fermentation of prebiotics produces short-chain fatty acids (SCFA) with numerous positive functions, including stimulating intestinal epithelial tissue, nourishing the intestinal cells, mucus production and secretion, increasing gut microbiome, and affecting their proper maturation and differentiation. Thus, it is believed that cashew nuts may offer the potential to modulate the microbiota composition as well as improve intestinal morphology and functionality [32,35,36,37]. However, current knowledge on the effect of cashew nuts on intestinal functionality, morphology, and microbiome is lacking [38,39,40,41,42,43,44,45,46].

*Gallus gallus* is an animal model widely used in scientific studies due to its biological and physiological characteristics that resemble those of humans, being recognized as an in vivo model of human nutrition [47,48,49]. This animal model has been applied to evaluate the functional effects of foods, mainly plant-based compounds, on gut health. Specifically, brush border membrane (BBM) functionality, prebiotic properties, and interaction with microbial population, as well as dietary mineral bioavailability [49,50]. This model has been widely used as it has a complex and dynamic intestinal microbiota influenced by genetics, environment, and the host’s diet. Its phylum-level microbiota is similar to that of humans (*Bacteroidetes*, *Firmicutes*, *Proteobacteria,* and *Actinobacteria*)—more than 85% of its intestinal gene sequences are homologous to human intestinal genes [51,52,53]; therefore, it shares significant genetic conservation with humans. It has rapid maturation, a well-characterized phenotype, and is very receptive to dietary manipulations [49,53]. In addition to these characteristics and advantages, *Gallus gallus* is one of the most studied models in biomedical research, occupying the third position, behind only rats and mice [54]. With all this, we have decided to use *Gallus gallus* as an animal model. Therefore, this study aimed to evaluate the effects of cashew nut soluble extract (CNSE) on intestinal BBM morphology, which means morphological parameters as Paneth cells, crypts, villi, and goblet cells, intestinal barrier as gene expression of occludin, AP, sodium-glucose transport protein 1 (SGLT-1), and mucin 2 (MUC2), inflammatory response as gene expression of NFκβ and IL-1β, and cecal bacteria populations, using the embryonic phase of *Gallus gallus* as the only external variable for the injected CNSE. Current investigation may contribute to the further understanding of the nutritional benefits of cashew nuts on gut health. 

## 2. Materials and Methods

### 2.1. Proteins and Dietary Fiber in Cashew Nut Flour and Cashew Nut Soluble Extract (CNSE)

Proteins and soluble and insoluble fibers were determined by the gravimetric-enzymatic method, AOAC International, using a total dietary fiber assay kit, Sigma^®^, San Luis, MO, USA [55]. More specifically, the analysis of protein content was performed in three repetitions and determined by the micro-Kjeldhal method, while total dietary fiber was performed in two repetitions.

### 2.2. Extract Preparation

The Brazilian Agriculture Research Corporation (EMBRAPA), Fortaleza-CE, Brazil, provided the cashew nut flower. The soluble extract was extracted as previously described [50,56]. To prepare the cashew nut extract, cashew nut flour samples were homogenized in distilled water in a concentration of 50 g of cashew nut flower for 1 L of distilled water at 60 °C, for 60 min, using a magnetic stirrer. The solution was filtered through a 600 μm screen to remove particulate matter. Then, the solution was placed in falcon tubes and centrifuged at 4000× *g* (4 °C) for 15 min. The supernatant collected was dialyzed (MWCO 12–14 kDa, Medicell International Ltd., London, UK) for 7 weeks against distilled water. The dialysate was lyophilized, which resulted in a fine off-white powder (cashew nut soluble extract).

### 2.3. Animals and Study Design

The protocol carrier out was approved by the Cornell University Institutional Animal Care and Use Committee (IACUC #2020-0077). The protocols were carried out in accordance with the relevant regulations and guidelines. About 40 fertile Cornish-cross broiler eggs were purchased from a commercial hatchery (Moyer’s chicks, Quakertown, PA, USA). The eggs were incubated under ideal conditions (37 ± 2 °C and 59.6 ± 2% humidity, embryonic days 0–18, 72.5 ± 1.8% hatch phase) until hatched at the Cornell University Animal Science poultry farm. CNSE in powder form was diluted in 18 Ω H_2_O to determine the concentrations necessary to maintain an osmolarity value (Osm) of less than 320 Osm to ensure that the viable embryos would not be dehydrated upon injection of the tested solution. The hatchability for the experiment was 34/40 eggs (85% survived). So, for the in vivo administration, 34 fertile eggs were weighed and randomly into 4 groups. On day 17 of incubation, candling was used to check fertilization and establish the injection spot. The spot was then sanitized, followed by 1 mL injected with a 21-gauge needle of the CNSE extracts and control (H_2_O). The four treatments were (1) no injection (n = 6), (2) 18 Ω H_2_O (n = 9), (3) 1% (10 mg/mL) CNSE (n = 10), and (4) 5% (50 mg/mL) CNSE (n = 9). After intra-amniotic administration, the injection spot was sealed with cellophane tape, and the eggs were incubated until hatch (day 21) [49]. Then, the birds were euthanized in a CO_2_ chamber, and the samples were collected. The duodenum and cecum were removed and placed in liquid nitrogen. The samples were then transferred to a −80 °C incubator until analysis. 

### 2.4. Gene Expression Analysis

#### 2.4.1. Extraction of Total RNA from Duodenum Tissue

Total RNA was extracted under RNase-free conditions from 30 mg of the duodenal tissue (n = 5). For that, we used Qiagen RNeasy Mini Kit (RNeasy Mini Kit, Qiagen Inc., Valencia, CA, USA) following the manufacturer’s protocol. The tissues in buffer RLT^®^, containing β-mercaptoethanol, were disrupted and homogenized with a rotor–stator homogenizer. The lysate was centrifuged at 8000× *g* (3 min) in a microcentrifuge (C2400-R, Labnet International Inc, Edison, NJ, USA). The supernatant was transferred to another tube with 70% ethanol and mixed immediately. A total of 700 μL of the sample was applied to an RNeasy mini column, centrifuged for 15 s at 8000× *g*, and the flow-through material was discarded. Then, the RNeasy columns were transferred to new collection tubes (2 mL), and 500 μL of buffer RPE^®^ was pipetted onto the RNeasy column, followed by centrifugation for 15 s at 8000× *g*. An additional 500 μL of buffer RPE was pipetted onto the RNeasy column and centrifuged at 8000× *g* (2 min). Total RNA was eluted in 50 μL of RNase-free water. RNA was quantified by absorbance at A 260/280. The integrity of the 18S ribosomal RNAs was verified by 1.5% agarose gel electrophoresis followed by ethidium bromide staining. DNA contamination was eliminated using a TURBO DNase treatment and removal kit from AMBION (Austin, TX, USA).

#### 2.4.2. Real-Time Polymerase Chain Reaction (RT-PCR)

cDNA was created from extracted RNA by a 20 µL reverse transcriptase (RT) reaction applying BioRad C1000 touch thermocycler using the Improm-II Reverse Transcriptase Kit (Catalog #A1250; Promega, Madison, WI, USA). For that, 1 µg of total RNA template, 2 mM of oligo-dT primers, and 10 µM of random hexamer primers were added to the vial. The ideal annealing temperature was 94 °C (5 min), and amplification was 60 min (42 °C)**,** followed by heat inactivation at 70 °C (15 min). The cDNA obtained was analyzed by Nanodrop (Thermo Fisher Scientific, Waltham, MA, USA) or stored at −80 °C until analysis, and the concentration was determined by measuring the absorbance at 260 nm and 280 nm with an extinction coefficient of 33 (for single-stranded DNA). The extent of genomic DNA contamination was estimated by an RT-PCR assay (real-time) for the reference gene samples.

#### 2.4.3. Primer Design

The Real-Time Primer Design Tool software (IDT DNA, Coralvilla, IA, USA) was utilized to design the primers based on six gene sequences from the Genebank database. The primer sequences (17–25-mier), amplicon length (restricted to 90–150 bp), and gene ID can be found summarized in Table 1. BLAST searches verified primer specificity against the genomic National Center for Biotechnology Information (NCBI) database. The *Gallus gallus* primer 18S rRNA served as a reference gene.

#### 2.4.4. Real-Time qPCR Design

cDNA (2 μL) was pipetted into a 96-well plate with 2× Bio-Rad SSO Advanced Universal SYBR Green Supermix (8 μL) (Cat #1725274, Hercules, CA, USA), followed by buffer, dNTPs, Taq polymerase, and dye. Both forward and reverse primers (Table 1) and cDNA (or water as control) were added to each PCR reaction. Each run had duplicates of 7 standard curve points. To prevent and eliminate any potential DNA contamination, a “no-template control” containing nuclear-free water was incorporated. DNA amplification was performed under the following conditions: an initial denaturation step at 95 °C for 30 s, 40 cycles of denaturation at 95 °C for 15 s, annealing temperatures varying according to Integrated DNA Technologies (IDT) for 30 s, and extension at 60 °C for 30 s, using a Bio-Rad CFX96 Touch (Hercules, CA, USA). Gene expression data were obtained by measuring the lowest cyclic product (Cp) values using the automated “second derivative maximum method”. The results were quantified against the standard curve, which was diluted at 1:10, and the reaction for each gene was run in duplicates. 

A graph showing the correlation between the Cq and log (10) concentrations was generated by the software, and the efficiencies were calculated as 10 (1/slope) (10). The specificity of the amplified real-time RT-PCR products was confirmed by analyzing the melting curves (ranging from 60 to 95 °C) after 40 cycles, which revealed several distinct products with specific melting temperatures [52,57]. 

### 2.5. Collection of Microbial Samples and Intestinal Contents DNA Extraction

Under sterile conditions, cecum samples (n = 5) were weighed (0.2 ± 0.02 g) and transferred to a 15 mL tube containing 9 mL of PBS (pH 7.4). Plastic beads were added to the tube, and the mixture was vortexed for 3 min. The tube was then centrifuged at 1000× *g* for 5 min, and the supernatant was collected and subjected to a second centrifugation step at 4000× *g* for 10 min. The buffer was discarded, and the pellet was washed twice with 1 mL of PBS before being stored at −20 °C until DNA purification. For purification, the pellet was treated with 50 mM EDTA (pH 8) and lysozyme (Sigma Aldrich Co., St. Louis, MO, USA) (10 mg/mL) at 37 °C. Bacterial DNA isolation was performed using a Wizard Genomic DNA purification kit according to the manufacturer’s protocol (Promega Corp., Madison, WI, USA).

### 2.6. Primers Design and PCR Amplification of Bacterial 16S rDNA Analysis

Primers for *Lactobacillus*, *Bifidobacterium*, *Clostridium, Escherichia coli,* and *L. plantarum* were used [58]. The universal primers were designed with the invariant region in the 16S rRNA of bacteria and were used as internal standards. The PCR products were separated by 2% agarose gel, stained with ethidium bromide, and quantified by Quantity One 1-D analysis software (Bio-Ra, Hercules, CA, USA). Each bacterium’s relative abundance was evaluated as previously described [50,52]. All products were expressed close to the content of the universal 16S rRNA primer product and proportions of each examined bacterial product. 

### 2.7. Morphological Examination of Duodenal Tissue

The intestinal morphology examination was conducted as previously described [51,59,60,61,62]. The duodenum samples per treatment group (n = 5) were preserved in 4% (*v*/*v*) formaldehyde solution, stabilized with phosphate buffer, dehydrated, cleaned, and embedded in paraffin. Four 5 μm sections of each sample were obtained and placed on a glass slide. The sections were deparaffinized in xylene, and the sample was rehydrated using a series of graded alcohol. The Alcian blue/periodic acid-Schiff (PAS) staining was applied in the slides, and the sample was assessed under a light microscope (BX3M series, Olympus, Waltham, MA, USA) using the CellSens Standard Software. Paneth cells were stained a light purple color. The number and diameter of Paneth cells were recorded, and the following morphometric measurements were evaluated: villus height and width; goblet cells number and diameter in villi and crypt; crypt depth; and goblet cell type (acid, neutral, and mixed) per villi and crypt. 

### 2.8. Statistical Analysis

The data in this paper are depicted as their mean values ± standard error. Experimental treatments and controls for intra-amniotic administration were assigned randomly after ensuring weight distribution to all groups. The Shapiro–Wilk test was used to assess data normality. One-way analysis of variance (ANOVA) or Kruskal–Wallis was used to analyze the results and *p*-values (*p* < 0.05), followed by post hoc Duncan or Dunn’s test. Statistical software IBM SPSS Statistics^®^, v25, was used for all analyses. 

## 3. Results

### 3.1. Concentration of Proteins, Total Dietary Fiber, and Fractions

The cashew nut soluble extract (CNSE) had a higher amount of proteins (41.65%) compared to cashew nut flour (21.50%) but a lesser amount of total fiber (9.82% vs. 22.36%), insoluble (9.29% vs. 21.33%), and soluble (0.53% vs. 1.03%) compared to the cashew nut flour (Table 2).

### 3.2. Body Weight

**Figure 1:**
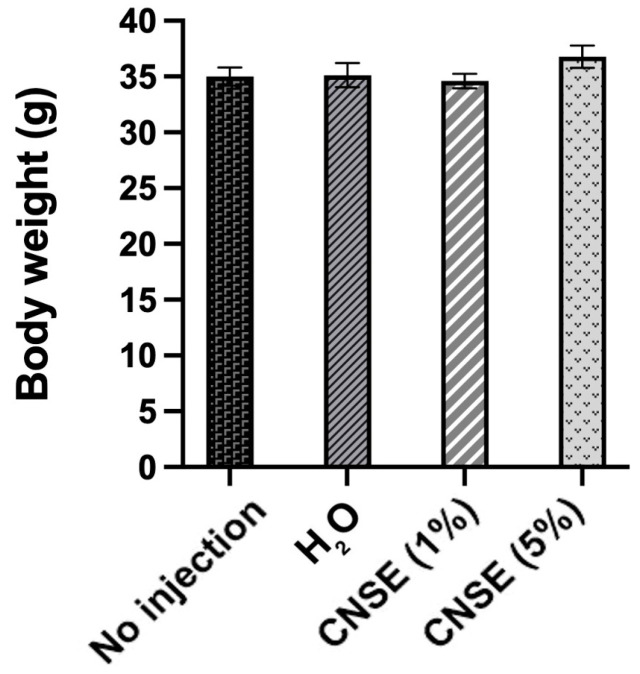
Body weight differences *between* groups. Mean and standard error of the mean (SEM) were evaluated using one-way ANOVA followed by Duncan test. CNSE: cashew nut soluble extract.

The body weight (g) was similar between groups with no statistical difference (*p*-value = 0.316) (Figure 1).

### 3.3. Effect of Cashew Nut Soluble Extract (CNSE) on Duodenal Morphological Parameters

CNSE promoted improvements in the morphometric parameters of the duodenum. At 5% and 1% CNSE, the Paneth cell number was higher compared to the controls (no injection and H_2_O) (Table 3). No difference was observed in Paneth cell diameter between groups (Table 3). In the crypts, the number of goblet cells (GCs) was smaller in CNSE treatments (1% and 5%), while GC diameter and depth were higher at 5% CNSE, all these compared to no injection (Table 4). In the villi, GC numbers were smaller in 1% and 5% concentrations compared to H_2_O injection; however, 1% was higher compared to no injection, and GC diameter was higher in both intervention groups compared to controls (no injection and H_2_O) (Table 4). Further, at a higher concentration of CNSE (5%), the villus surface area was larger compared to other groups (Table 4). 

Related to the type of GC (acid, neutral or mixed) in villi, the acid was smaller in treatment groups compared to control groups (no injection and H_2_O), the neutral was smaller in all groups compared to no injection, and the mixed type was higher in CNSE treatments (being higher at 1% compared to 5%) compared to the control groups (no injection and H_2_O) (Table 5). Regarding the type of GC in the crypt, neutral and mixed were smaller at 5% compared to H_2_O and all other groups, respectively (Table 5). No difference was observed for acid GC per crypt (Table 5).

**Figure 2:**
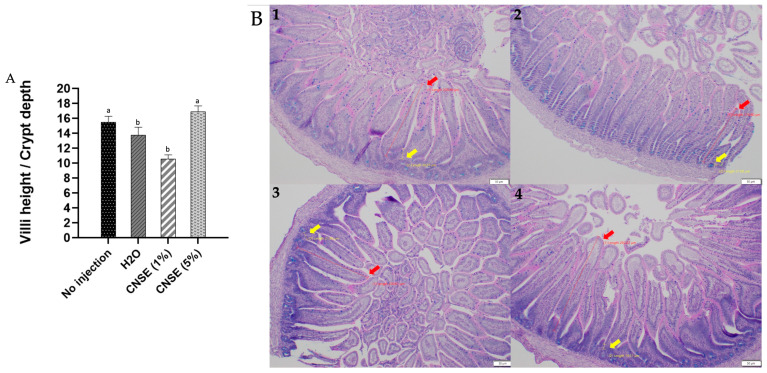
(**A**) Villi height/crypt depth ratio between groups. Mean and standard error (SEM) were used. Superscript alphabets (^a,b^) not indicated by the same letter in the figure means that there was a statistical difference between groups (*p* < 0.05) according to one-way ANOVA with post hoc Duncan test, letter “^a^” represents the highest value. (**B**) Villi height (red arrow) and crypt depth (yellow arrow) in each group is represented through the intestinal morphology analysis. 10× sizing was used in each measurement. 1: No injection; 2: H_2_O; 3: 1% cashew nut soluble extract (CNSE); 4: 5% CNSE.

When evaluating the villi height/crypt depth ratio, the value was higher in 5% CNSE (16.89 ± 0.76) than in other groups (no injection: 15.46 ± 0.81; H_2_O: 13.76 ± 1.04; 1% CNSE: 10.55 ± 0.56) (Figure 2).

### 3.4. Effect of Cashew Nut Soluble Extract on the Abundance of Intestinal Bacterial Populations 

**Figure 3:**
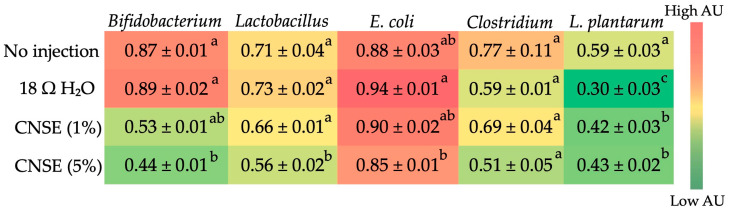
Heatmap of the difference between the bacterial population in cecum by intra-amniotic administration of injected CNSE in different concentrations (10 mg/mL; 50 mg/mL). Superscript alphabets (^a–c^) not indicated by the same letter in the same column means that there was a statistical difference between groups (*p* < 0.05) according to one-way ANOVA or Kruskal–Wallis, followed by post hoc tests. When there is a statistical difference between groups, the superscript alphabets (^a–c^) are represented in descending order of the values (letter “^a^” represents the highest value, while letter “^c^” is the lowest). CNSE: cashew nut soluble extract.

In relation to bacterial population in cecum contents, the intervention groups that received the CNSE at a concentration of 50 mg/mL (5%) had a lower abundance of *Bifidobacterium* compared to control groups and *Lactobacillus* compared to all other groups. For *E. coli*, the group that received 5% of the CNSE had a lower abundance than the group that received H_2_O. For *Clostridium* spp., there was no statistical difference between groups. Regarding *L. plantarum*, the intervention groups (1% and 5% of the CNSE) had higher abundance when compared to the group that received H_2_O and lower than the no-injection group (Figure 3).

### 3.5. Effect of Cashew Nut Soluble Extract on Gene Expression of Intestinal Barrier Proteins and Inflammatory Biomarkers

**Figure 4:**
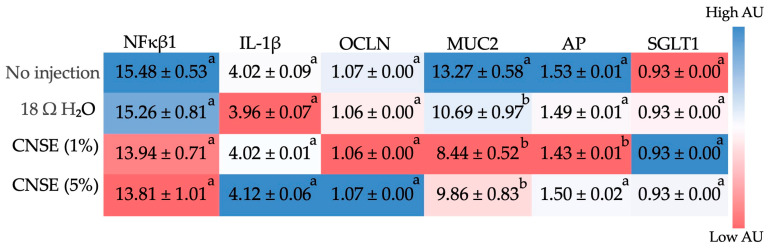
Heatmap of the difference between duodenal gene expression by intra-amniotic administration of injected CNSE in different concentrations (10 mg/mL; 50 mg/mL). Superscript alphabets (^a,b^) not indicated by the same letter in the same column means that there was a statistical difference between groups (*p* < 0.05) according to one-way ANOVA or Kruskal–Wallis, followed by post hoc tests. When there is a statistical difference between groups, the superscript alphabets (^a,b^) are represented in descending order of the values (the letter “^a^” represents the highest value). NFκβ1: nuclear factor kappa beta; IL-1β: interleukin 1 beta; OCLN: occludin; MUC2: mucin 2; AP: aminopeptidase; SGLT1: sodium-glucose cotransporter 1; CNSE: cashew nut soluble extract.

NFκβ1 and IL-1β are pro-inflammatory genes, and no statistical difference was identified in these genes between groups. Regarding those genes related to the intestinal barrier, which are occludin (OCLN), mucin 2 (MUC2), aminopeptidase (AP), and sodium-glucose cotransporter 1 (SGLT-1), no difference was observed between groups for OCLN and SGLT1. In contrast, for MUC2, there was a reduction in the groups that received H_2_O, 1%, and 5% CNSE compared to the no-injection group. Besides this, an upregulation in the AP in 5% CNSE compared to the 1% was observed (Figure 4).

## 4. Discussion

Although cashew nut is an excellent dietary source of dietary fiber [16], studies describing the dietary effects of cashew nuts on intestinal functionality, morphology, and microbiome, are scarce. To our knowledge, the current study is the first to investigate this. 

The microbial analysis indicated that the groups that received CNSE had decreased abundance of *Bifidobacterium*, *Lactobacillus*, and *E. coli* compared to the control groups. Further, there was no statistical difference between treatment groups for *Clostridium* spp. In the case of *L. plantarum*, there was a lower abundance in the intervention groups compared to the no-injection group and a greater abundance when compared to the H_2_O injection. *Lactobacillus* and *Bifidobacterium* are known as probiotics, whereas *Clostridium* is potentially pathogenic, and *E. coli* depending on the strain, can be either pathogenic or beneficial [49].

Of the total dietary fiber that was measured in the CNSE used in this study, 9.29% were insoluble dietary fibers, while 0.53% were soluble dietary fibers. Soluble dietary fibers are rapidly fermented, while insoluble dietary fibers are slowly or only partially fermented [63]. This may explain the reduced fermentation in the CNSE treatment groups. *Lactobacillus*, *Bifidobacterium,* and *L. plantarum* belong to the lactic acid bacteria groups and produce lactate through fermentation [64]. Similarly, in a study that assessed the soluble extract of chia seed, assessed concentrations of 1% and 5% of the soluble section of chia also showed a reduction in *Bifidobacterium* and *E. coli*, and at a 5% concentration, there was a *Lactobacillus* reduction [65]. Chia flour (2.89 g/100 g soluble; 30.47 g/100 g insoluble) [66] and cashew nut flour (1.03 g/100 g soluble; 21.33 g/100 g insoluble) have similar contents of fiber fractions, but when we compare the fiber fractions of the soluble extract, the CNSE presents much fewer amounts of soluble fibers (0.53 g/100 g vs. 19.68 g/100 g) and insoluble (0.47 g/100 g vs. 23.53 g/100 g) compared to the soluble extract of chia [65]. Previously, Dias et al. (2019) used 5% of the soluble extract of carioca beans and documented a reduction in *Bifidobacterium* at this concentration compared to the control groups [52]. On the other hand, a study that used a soluble extract of chickpeas and lentils showed an increased abundance of *Bifidobacterium* and *Lactobacillus* in the 5% extraction treatment groups, compared to the control groups (*p* < 0.05) [56]. However, it is important to note that the soluble/insoluble dietary fiber ratios in chickpeas (0.22) and lentils (0.23) are higher compared to the soluble/insoluble dietary fiber ratios in cashew nuts (0.05) and chia (0.09). Further, chickpea has 3.5 g/100 g of soluble fiber and 15.8/100 g of insoluble, and lentil has 3.1 g/100 g of soluble and 13.6 g/100 g of insoluble [67]. This might be a possible explanation of why the amount of 5% of chickpea and lentil extracts was sufficient to increase the relative abundance of these beneficial bacteria, given that these foods have a higher soluble/insoluble dietary fiber ratio. Wang et al. (2019) evaluated wheat bran, which is rich in insoluble dietary fiber (35–48.4 g/100 g) and arabinoxylan (22–30 g/100 g) [68], and showed that higher amounts of wheat bran extract (10%) promote higher abundance of *Bifidobacterium* and *Lactobacillus* compared to a lower concentration (5%) [69]. Altogether, these findings further reinforce the hypothesis that a higher amount of cashew nut soluble extract may be necessary to increase the concentration of these beneficial bacterial populations.

It is important to emphasize that *L. plantarum* competes with Gram-negative or other potential pathogens (i.e., *E. coli*) on receptor sites at the mucosal cell surfaces in the gastrointestinal tract (GIT) [70]. In the present study, the 5% CNSE treatment group had an increased abundance of *L. plantarum* (compared to the H_2_O injection group) and a reduced abundance of *E. coli*, which can perhaps be explained by the competition of these bacteria on the same receptor. Another possible hypothesis to explain the reduction in pathogenic bacterial abundance is associated with the increased number of Paneth cells. Paneth cells are found in the intestinal villi crypts and produce and secrete various antimicrobial peptides (AMPs), which are essential for defense against intestinal pathogens [71]. In the current study, it was demonstrated that in treatment groups where the Paneth cell number increased, the *E. coli* and *Clostridium* decreased. It is, therefore, possible that the increased production of AMPs by Paneth cells generated by the CNSE contributed to the reduced abundance of bacterial populations. In addition, a study that used inulin (which is a soluble fiber) showed that, unlike cellulose (which is insoluble), inulin increased the level of Paneth cells [72]. In addition to fibers, CNSE showed high concentrations of proteins, and these also exert effects on the composition of the microbiota. The protein source is a very important determinant for the utilization of intestinal bacteria. However, compared to proteins from plant-based food, those from animal-based food sources seem to have better effects on the microbiota, first because protein digestibility from animal sources is higher, and second, because digestion of plant proteins may be limited by the presence of antinutritional factors found in plants [73]. As demonstrated in a study, rats fed with protein from animal-based food increased *Lactobacillus* and/or *Bifidobacterium* compared to those fed from plant-based food [74,75,76,77]. Thus, despite the high protein content found in the CNSE, as the source of this protein is a plant-based food, this may also have contributed to the non-growth of beneficial bacteria. 

Regarding gene expression, although there were no statistical differences in investigated inflammation-related genes, the results suggested a trend toward a reduction in NFκβ1 gene expression in the CNSE groups compared to the control groups. It is possible that the lower abundance of beneficial bacteria (*Lactobacillus* and *Bifidobacterium*) in the CNSE groups has not been able to generate enough anti-inflammatory effects to be significant. This is because these bacterial populations produce lactate, which is often associated with immunomodulating properties via suppression of the LPS/Toll-like receptor 4 signaling pathway [78]. Lactate and acetate can also be converted to butyrate by intestinal bacteria, which supplies energy to colonic epithelial cells, maintaining gut barrier functions and modulating the immune system in an anti-inflammatory manner [79]. Thus, these metabolites have anti-inflammatory action. Therefore, less concentrated bacteria and low fermentation and production of these metabolites were insufficient to make this anti-inflammatory effect significant in the CNSE groups compared to the control. In contrast, the upregulation in IL-1β gene expression at the 5% CNSE group compared to the control groups may have been due to competition between *L. plantarum* and *E. coli*, producing toxins that are responsible for generating inflammation [80,81]. This hypothesis is confirmed by the increased number of Paneth cells in this group since these cells are needed to produce antimicrobials [82]. Further, this is supported by the fact that IL-1β is produced by immune cells (i.e., monocytes and macrophages) and non-immune cells (i.e., endothelial and epiderm cells) in response to different stimuli, including microbes, bacterial lipopolysaccharides (LPS), and cytokines [83]. 

Regarding the BBM functional proteins, MUC2 is expressed and secreted by GC, and it is the prominent intestinal gel-forming mucin of the mucus layer of the small intestine, creating a protective mucus layer against bacterial invasion [84,85]. They are also involved in the immunoregulation and intestinal digestive and absorptive capabilities [86]. In this study, there were fewer GC numbers in the intervention groups, which may have impacted mucin production and secretion and consequently downregulated the MUC2 gene; since there were a small number of cells, there would be less production of mucins. In addition, intestinal bacteria play an essential role in mucin secretion, as *Lactobacillus*, *Bifidobacterium*, and *L. plantarum* species could increase the synthesis and secretion of mucins [81,87,88]. Duangnumsawang et al. (2021) showed that the lack of gut bacteria in germ-free chickens led to a reduction in the number and density of GC, as well as a decrease in MUC2 expression [87]. Other studies showed that germ-free animals exhibited a decrease in GC size and number with a consequent reduction in mucus layer thickness, indicating a reduction in mucus production [89,90]. The lower concentration of bacteria (*Lactobacillus*, *Bifidobacterium*, and *L. plantarum*) in the CNSE groups may also have contributed to a lower number of GC, consequently lower mucin secretion and reduced MUC2 gene expression.

The expression of the amino peptidase (AP) gene was dose-dependently upregulated in CNSE. AP is involved in protein and peptide degradations; it cleaves amino acids from the *N*-terminus of peptides and provides substrates for the amino acid transporters. Proteins are soluble in water. As demonstrated in the Results section, the amount of protein in CNSE was much higher (41.65%) than in cashew nut flour (21.50%). Therefore, AP could act in the hydrolysis of these proteins and peptides, increasing the AP gene expression [91]. One of the most abundant amino acids in cashew nuts is glutamic acid [21], which is the primary energy source for intestinal epithelial cells [92]. No differences were found in OCLN and SGLT1 expression, as the values were practically the same between groups. OCLN is regarded as one of the most critical tight junction-associated structural proteins in the intestine and plays a vital role in keeping the physical barrier of the intestinal mucosa [93]. Similar results were described in previous studies using the same in vivo model [52,57,94,95]. The authors highlighted that the *Gallus gallus* model has a limited ability to digest and absorb nutrients before hatch [71,96], as AP and SGLT1 are biomarkers of BBM digestive and absorptive functions; perhaps for that reason, a longer intervention time would be necessary to be able to observe more significant differences in BBM functionality.

The intestinal morphology results showed an increase in the crypt GC diameter and depth in the group that received 5% CNSE compared to the no-injection group. These results may be associated with a reduction in GC number to maintain homeostasis. As this group had few numbers of GC, there were an increase in the diameter and depth of these existing cells. Deeper crypts may indicate a renewal of intestinal epithelial cells, positively affecting intestinal absorption and secretion function [97]. Dietary fibers mechanically stimulate the intestinal epithelium to secrete mucus, thus stimulating the secretion of water and mucous as a defense mechanism to protect [70]. Zou et al. (2018) demonstrated that inulin (fermentable dietary fiber), but not cellulose (not fermentable fiber), was able to reverse reductions in colon mass and crypt length, gut atrophy, reduced enterocyte proliferation, and microbiota encroachment from a high-fat diet (HFD) [58]. In addition, the high concentration of protein present in the extract may also have affected the number of goblet cells. One study showed that higher amounts of protein (53%) compared to lower doses (14%) showed a lower number of GC at the epithelial surface [98].

Regarding the villi, the present study showed an increase in the villi surface area in the 5% CNSE treatment group compared to others. The longer the length of villi, the greater the absorption of nutrients, and this is dependent on the diet provided. This may have been attributed to high protein concentrations in the 5% CNSE as amino acids, mostly glutamine (which comes from glutamic acid), one of the main fuels for the small intestine mucosa, as they provide the energy required for intestinal ATP-dependent metabolic processes, such as active nutrient transport and high rates of intracellular protein turnover [99]. Besides the protein, this finding could also be attributed to soluble dietary fibers that caused an increase in the proliferation of enterocytes, which leads to hyperplasia/hypertrophy of these cells and, consequently, growth in villi surface area. Thus, improving the absorptive and digestive capacity of the villi [65]. This is supported by current findings, as the 5% CNSE group showed higher values in the villi height/crypt depth ratio relative to other groups. The relationship between these two markers indicates intestinal health, as the appropriate proportion between villus height and crypt depth are important indicators of gut development and animal health and, as such, influence nutrient digestion and absorption [97]. Shorter villi reduce the surface area as well as deeper crypt lead to a higher secretion of digestive enzymes, which consequently decreases nutrient absorption [97,100,101].

Related to the distribution of mucin types (acid and neutral) in GC, they can be affected by host factors (inflammatory markers, neurotransmitters, or hormones) and external factors (commensal bacteria, pathogens, dietary nutrients, or pre/probiotics) [102]. In this study, we demonstrated less acid and neutral GC per villi (where the mucus is produced) in CNSE groups. This result is because these groups had less abundance of bacteria compared to the control groups. This hypothesis can be reinforced based on a study that compared conventionally raised (CR) animals and germ-free animals (GF) and showed that GF animals displayed less neutral mucin and sulfated (i.e., acid) mucin compared to CR [103]. Lastly, it is essential to highlight that this was the first study to evaluate the cashew nut soluble extract on intestinal health in vivo, and subsequent studies are needed. However, the results demonstrated in this study contribute to a better understanding of cashew nuts’ benefits on intestinal health through their action on different parameters such as morphology, functionality, and microbiota. Understanding all these factors contributes to differentiation in future studies, mainly clinical trials.

## 5. Conclusions

Cashew nut soluble extract improved gut health by promoting benefits on morphological parameters as it increased villi surface area, villi height/crypt depth ratio, and intestinal functionality by upregulating AP gene expression. All these parameters are essential for improving digestive and absorptive capacity. In addition, cashew nut soluble extract was beneficial for acting in defense against intestinal pathogens by increasing Paneth cell number. Thus, serving as a preliminary step to provide a greater understanding of the potential of cashew nuts to promote gut health. As this was the first study to assess the effects of cashew nuts on gut health in vivo, more studies are needed, mainly using higher concentrations of the extract and long-term studies to elucidate additional potential health benefits and mechanisms of action. As well, future studies using next-generation sequencing (NGS) methods can also help to identify greater bacteria diversity from the consumption of cashew nut soluble extract.

## Figures and Tables

**Table 1:** DNA sequences of primers used.

Analyte	Forward Primer (5′→3′)	Reverse Primer (5′→3′)	Base Pair	GI Identifier
Intestinal Barrier				
AP	CGTCAGCCAGTTTGACTATGTA	CTCTCAAAGAAGCTGAGGATGG	138	45,382,360
SGLT1	GCATCCTTACTCTGTGGTACTG	TATCCGCACATCACACATCC	106	8,346,783
MUC2	CCTGCTGCAAGGAAGTAGAA	GGAAGATCAGAGTGGTGCATAG	155	423,101
OCLN	GTCTGTGGGTTCCTCATCGT	GTTCTTCACCCACTCCTCCA	124	396,026
Inflammatory Response				
NF-κβ	CACAGCTGGAGGGAAGTAAAT	TTGAGTAAGGAAGTGAGGTTGAG	100	2,130,627
IL-1β	TCATCCATCCCAAGTTCATTCA	GACACACTTCTCTGCCATCTT	105	395,872

AP: amino peptidase; SGLT1: sodium-glucose transport protein 1; MUC2: mucin 2; OCLN: occludin; NF-κβ: nuclear factor kappa beta; IL-1β: interleukin 1 beta.

**Table 2:** Content of proteins, total dietary fiber, and fractions in cashew nut flour and soluble extract.

	Proteins	Fibers		
		Total	Insoluble	Soluble
Cashew nut flour (g/100 g)	21.50 ± 0.51	22.36 ± 0.05	21.33 ± 0.40	1.03 ± 0.45
Cashew nut soluble extract (g/100 g)	41.65 ± 0.18	9.82 ± 0.42	9.29 ± 0.47	0.53 ± 0.05

Data are presented as mean and standard deviation.

**Table 3:** Effect of intra-amniotic administration of CNSE on Paneth cell between groups.

Groups	Paneth Cell	
	Number	Diameter (μM)
No injection	1.94 ± 0.08 ^bc^	1.45 ± 0.04 ^a^
18 Ω H_2_O injection	1.68 ± 0.06 ^b^	1.40 ± 0.04 ^a^
CNSE (1%)	2.43 ± 0.11 ^a^	1.42 ± 0.03 ^a^
CNSE (5%)	2.39 ± 0.12 ^ac^	1.34 ± 0.04 ^a^

Superscript alphabets (^a–c^) not indicated by the same letter in the same column means that there was a statistical difference between groups (*p* < 0.05) according to one-way ANOVA or Kruskal–Wallis followed by post hoc tests. When there is a statistical difference between groups, the superscript alphabets (^a–c^) are represented in descending order of the values (letter “^a^” represents the highest value, while letter “^c^” is the lowest); CNSE: cashew nut soluble extract.

**Table 4:** Effect of intra-amniotic administration of CNSE on crypt and villi measurements between groups.

**Groups**	**Crypt**		
	**GC Number**	**GC Diameter (μM)**	**Depth (μM)**
No injection	12.67 ± 0.55 ^a^	2.92 ± 0.05 ^b^	14.02 ± 0.50 ^b^
18 Ω H_2_O injection	10.95 ± 0.62 ^b^	3.13 ± 0.05 ^a^	16.00 ± 0.65 ^ab^
CNSE (1%)	9.72 ± 0.41 ^b^	3.12 ± 0.06 ^ab^	15.80 ± 0.56 ^ab^
CNSE (5%)	9.39 ± 0.42 ^b^	3.23 ± 0.06 ^a^	17.10 ± 0.69 ^a^
	**Villi**		
	**GC number**	**GC Diameter (μM)**	**Surface Area (mm²)**
No injection	24.68 ± 0.74 ^c^	2.46 ± 0.06 ^b^	11668.81 ± 446.06 ^b^
18 Ω H_2_O injection	38.38 ± 0.91 ^a^	2.20 ± 0.05 ^c^	8248.42 ± 364.05 ^c^
CNSE (1%)	33.41 ± 0.81 ^b^	2.81 ± 0.07 ^a^	8341.78 ± 342.77 ^c^
CNSE (5%)	25.59 ± 0.82 ^c^	2.79 ± 0.10 ^a^	14491.08 ± 505.97 ^a^

Superscript alphabets (^a–c^) not indicated by the same letter in the same column means that there was a statistical difference between groups (*p* < 0.05) according to one-way ANOVA or Kruskal–Wallis followed by post hoc tests. When there is a statistical difference between groups, the superscript alphabets (^a–c^) are represented in descending order of the values (letter “^a^” represents the highest value, while letter “^c^” is the lowest). GC: goblet cell. CNSE: cashew nut soluble extract.

**Table 5:** Effect of intra-amniotic administration of CNSE on GC type in villi and crypt between groups.

Groups	GC per Villi			GC per Crypt		
	Acid	Neutral	Mixed	Acid	Neutral	Mixed
No injection	15.28 ± 0.71 ^b^	0.79 ± 0.13 ^a^	8.68 ± 0.57 ^c^	8.53 ± 0.46 ^a^	0.41 ± 0.06 ^ab^	3.73 ± 0.27 ^a^
18 Ω H_2_O injection	26.71 ± 1.12 ^a^	0.10 ± 0.04 ^b^	11.57 ± 0.66 ^b^	7.88 ± 0.51 ^a^	0.50 ± 0.07 ^a^	2.58 ± 0.21 ^bc^
CNSE (1%)	12.13 ± 0.54 ^c^	0.24 ± 0.09 ^b^	21.03 ± 0.75 ^a^	6.93 ± 0.30 ^a^	0.29 ± 0.06 ^ab^	2.63 ± 0.17 ^ab^
CNSE (5%)	10.87 ± 0.66 ^c^	0.57 ± 0.14 ^b^	14.16 ± 0.72 ^b^	7.34 ± 0.35 ^a^	0.30 ± 0.07 ^b^	1.76 ± 0.14 ^c^

Superscript alphabets (^a–c^) not indicated by the same letter in the same column means that there was a statistical difference between groups (*p* < 0.05) according to one-way ANOVA or Kruskal–Wallis followed by post hoc tests. When there is a statistical difference between groups, the superscript alphabets (^a–c^) are represented in descending order of the values (letter “^a^” represents the highest value, while letter “^c^” is the lowest). GC: goblet cell. CNSE: cashew nut soluble extract.

## Data Availability

Not applicable.
